# Adherence to gestational diabetes screening guidelines: Retrospective audit in three public hospitals in Ethiopia

**DOI:** 10.1371/journal.pone.0357102

**Published:** 2026-08-25

**Authors:** Meselech Assegid Roro, Melaku Taye Amogne, Mistire Wolde Gebre, Elizabeth C. Rhodes, Leslie C. M. Johnson, Rebecca Gary, Saria Hassan, Rosette Chakkalakal, Workeabeba Abebe

**Affiliations:** 1 Department of Reproductive, Family and Population Health, School of Public Health, College of Health Sciences, Addis Ababa University, Addis Ababa, Ethiopia; 2 Department of Internal Medicine, School of Medicine, College of Health Sciences, Addis Ababa University, Addis Ababa, Ethiopia; 3 Department of Medical Laboratory Science, School of Biomedicine and Medical Laboratory Science, College of Health Sciences, Addis Ababa University, Addis Ababa, Ethiopia; 4 Hubert Department of Global Health, Rollins School of Public Health, Emory University, Atlanta, Georgia, United States of America; 5 Emory Global Diabetes Research Center, Woodruff Health Sciences Center and Emory University, Atlanta, Georgia, United States of America; 6 Department of Family and Preventive Medicine, School of Medicine, Emory University, Atlanta, Georgia, United States of America; 7 Nell Hodgson Woodruff School of Nursing, Emory University, Atlanta, Georgia, United States of America; 8 Department of Medicine, Emory School of Medicine, Emory University, Atlanta, Georgia, United States of America; 9 Department of Pediatrics and Child Health, School of Medicine, College of Health Sciences, Addis Ababa University, Addis Ababa, Ethiopia; Zhejiang University School of Medicine Women’s Hospital, CHINA

## Abstract

**Introduction:**

Gestational diabetes mellitus (GDM) is a major contributor to adverse perinatal outcomes. Most guidelines recommend universal GDM screening using standardized testing protocols, yet implementation in low- and middle-income countries remains limited. This study evaluated GDM screening uptake and guideline-concordant testing in routine antenatal care in Addis Ababa, Ethiopia.

**Methods:**

We conducted a facility-based, cross-sectional study of pregnant women receiving antenatal care in three public hospitals in Addis Ababa, Ethiopia. A sample of 1,050 antenatal records was abstracted for women who were ≥28 weeks of gestation and/or had delivered during March to May 2025. Using a structured checklist, we extracted maternal and obstetric characteristics, documentation of any GDM screening, risk-based indications for screening, gestational age at testing, screening methods, and GDM diagnosis. We described uptake and adherence to guideline-recommended GDM screening. Logistic regression was used to estimate crude odds ratios (COR) and adjusted odds ratios (AOR) with 95% confidence intervals (CI) for factors associated with being screened.

**Results:**

The mean (±SD) age of participants was 27.7 (± 5.0) years. Approximately one-third (29.4%) were pregnant for the first time. Overall, 139/1050 women were screened for GDM, yielding a screening rate of 13.2%. Median gestational age at screening was 34 weeks [IQR 30–37]; only 8.6% of screened women were tested in the recommended 24–28-weeks window. The oral glucose tolerance test was the predominant method (87.8%). Screening was almost exclusively risk-based (99.3%), where testing was prompted by clinical indications including polyhydramnios, advanced maternal age, and a prior history of macrosomic birth. Among screened women, 57/139 (41.0%) were diagnosed with GDM. In adjusted analysis, facility site (hospital C), older maternal age, higher parity and earlier gestational age at first antenatal care were independently associated with higher odds of being screened.

**Conclusion:**

In three public hospitals in Addis Ababa, GDM screening uptake was low. The screening practice was also delayed, and risk-based. Being screened was associated with hospital site, older maternal age, higher parity, and earlier first antenatal care attendance. These findings suggest addressing patient-level factors and facility-tailored implementation strategies to achieve universal, timely GDM screening in routine antenatal care workflows.

## Introduction

Gestational diabetes mellitus (GDM) is one of the most common medical complications of pregnancy worldwide. Recent estimates from the International Diabetes Federation (IDF) showed that one in five live births (19.7%) are affected by hyperglycemia during pregnancy, with GDM accounting for the majority (79.2%) of these cases [1]. The burden is concentrated in low- and middle-income countries (LMICs), which account for almost 90% of affected pregnancies [1,2]. This reflects marked disparities in GDM prevalence and treatment in populous LMICs where resources for and access to prenatal care are limited [3].

GDM is an important driver of adverse maternal and perinatal outcomes. It has been associated with pregnancy complications such as preeclampsia, operative delivery, macrosomia, stillbirth and neonatal complications. GDM is also associated with increased long-term risk of type 2 diabetes and cardiovascular diseases for both the mother and child [4–9].

Early identification of GDM enables timely lifestyle and pharmacologic intervention that reduce adverse pregnancy outcomes such as reduction in cesarean births, birth injuries, neonatal intensive care unit admissions, stillbirth and improve long-term cardiometabolic trajectories [10–12]. Accordingly, major international bodies, including the World Health Organization (WHO), the International Federation of Gynecology and Obstetrics (FIGO) and the American Diabetes Association (ADA), recommend universal GDM screening for all pregnant women, typically using a one-step 75-gram oral glucose tolerance test (OGTT) at 24–28 weeks’ gestation [13–16].

Many high-income countries have operationalized these recommendations into clinical pathways and achieve very high screening coverage, over 90% [17–19]. In contrast, in most Sub-Saharan African countries, GDM screening remains selective (risk-based), with heterogeneous testing methods and timing, and large proportions of women are never screened at all [20,21]. In Cameroon, one study found that the GDM screening rate was only 22%, and among those screened, only about 5% had been tested during the recommended 24–28-weeks gestational window [20]. In Tanzania, an urban hospital-based study reported GDM screening coverage of 29.9%, with substantial variation between facilities (21.5% versus 38.1%) and limited use of OGTT (only 3.4%) as the screening method [21]. A survey of antenatal healthcare providers in Nigeria also found that 68% of the clinicians reported performing universal screening but only 22.1% reported using OGTT [22].

Available African evidence suggests that GDM screening is shaped by maternal risk, access to care, provider practice, and health-system readiness. In Tanzania, screening was associated with prior macrosomic baby, pregnancy-induced hypertension, and awareness of GDM [21]; in Ghana, return for scheduled GDM testing was associated with age, education, employment, residence, level of care, and test acceptability [23]; and in Nigeria, provider and facility characteristics influenced universal versus risk-based screening practice [22]. Qualitative evidence from Ethiopia [24] and an African systematic review [25] further identify late ANC attendance, lack of screening guidelines, limited provider knowledge, shortages of trained staff, inadequate equipment and consumables, costs, and poor acceptability of OGTT as barriers to GDM screening.

In Ethiopia, GDM has emerged as a public health challenge. In pooled evidence of studies from Ethiopia, a recent systematic review and meta-analysis estimated a GDM prevalence of approximately 12%, although individual studies report a wide range depending on diagnostic criteria, testing strategy, and regions, from 6.28% to 12.34% [26]. Although Ethiopia formally endorsed the 2013 WHO recommendation for universal GDM screening, national antenatal care (ANC) guidance has not consistently translated this policy into an operational, standardized screening pathway [27]. The practice continues to be largely selective screening [24], leaving a significant number of women undiagnosed and at risk of pregnancy complications. Despite this high burden of GDM in Ethiopia, there is a limited evidence on GDM screening practice in routine ANC. This represents a major implementation knowledge gap: without baseline local data on screening practice, efforts to improve GDM detection risk being poorly targeted.

To address this gap, we conducted a retrospective chart review in three public hospitals in Addis Ababa to generate context-specific evidence on GDM screening uptake and adherence to guideline-recommended practice. Specifically, we aimed to quantify the proportion of pregnant women who received any GDM screening, characterize screening timing, and testing methods.

## Methods

### Study design and setting

We conducted a cross-sectional record review in three public hospitals in Addis Ababa, Ethiopia: Tikur Anbessa Specialized Hospital, Gandhi Memorial Hospital and Abebech Gobena MCH Hospital from March to May, 2025. These tertiary care hospitals were purposively selected because they provide dedicated maternal health services with high ANC client volume, including GDM care. As such, these public facilities would ideally represent settings where guideline-recommended GDM screening would be carried out more reliably compared to lower-level settings in Ethiopia’s three-tier health system.

### Study population and eligibility

The source population comprised of pregnant women who had ANC follow-ups at the three hospitals. The study population included pregnant women whose ANC records were available in the participating hospitals and who met the of the following criteria during the study period: 1) gestational age of  ≥ 28 weeks or 2) delivery in one of the study hospitals between March and May 2025. Women were eligible if they had attended at least two ANC visits in the same facility during the index pregnancy. Women with known pre-existing type 1 or type 2 diabetes were excluded.

### Sample size and sampling procedure

The sample size was estimated using the StatCalc sample size calculator in Epi Info version 7 for a single-population proportion formula. Assuming a 95% confidence level and a 5% margin of error, and taking the highest GDM screening rate of 29.9% from a sub-Saharan African study [21] as the expected proportion, the minimum required sample size was 323. Because the number of eligible ANC records during the three-month study period was manageable, we used a census approach and abstracted all eligible records. A total of 1,050 (350 per facility) eligible electronic medical records (EMRs) were included during the study period.

### Data collection and variables

Data were abstracted using a structured, pre-tested chart review checklist. The checklist captured socio-demographic and obstetric characteristics, and GDM screening variables (documentation of any GDM tests, time of screening, type of screening test, test results and risk factors prompting testing). The guideline-recommended test for GDM screening is the 75 g oral glucose tolerance test (OGTT), performed after an oral glucose load, with fasting and post-load glucose measurements, and offered universally at 24–28 weeks’ gestation.

Data were collected from the EMRs by trained data collectors (4 General Practitioners and 1 Health Officer, a provider with BSc in Public Health). The hospitals’ laboratory information systems were also cross-checked for records of OGTT, fasting blood sugar (FBS), random blood sugar (RBS) or hemoglobin A1c (A1c) requests and results during the index pregnancy. ODK version 1.25.2 software was used to collect the data along with the KoboToolbox server to store the collected data securely. Data were accessed for the research purposes between 19/06/2025 and 29/08/2025.

Our primary outcome was GDM screening uptake, defined as evidence of one or more of laboratory tests (OGTT, FBS, RBS, or A1c) to assess glycemia during pregnancy. The secondary outcome was adherence to GDM screening guideline recommendation, defined by a set of indicators: who is screened, when screened and what screening test. We also assessed GDM diagnosis rate, and the risk-based indications for screening among women who were screened.

### Data quality assurance

Data quality was ensured through training of data collectors, use of a standardized abstraction checklist, and regular supervisory review. Data collectors were trained on the study objectives, eligibility criteria, variable definitions, and use of chart abstraction form. The abstraction tool was reviewed by the study team multiple times and pilot-tested. During data collection, supervisors reviewed completed forms regularly for completeness and adherence to variable definitions. Range checks were applied to key variables during data collection. Duplicate records were checked using patient identifiers.

We triangulated multiple data sources before classifying screening status. For each eligible woman, data collectors reviewed the EMR (has both antenatal care records, and delivery/maternity records), and the hospital laboratory information system.

### Statistical analysis

The ODK collected data were validated and exported to SPSS 25 for analysis. Data were systematically cleaned before analysis. Range and consistency checks were applied to key variables. For continuous covariates with few missing values, we applied single imputation using the facility-specific median or mean, assuming missingness was approximately random within facilities.

We summarized continuous variables using means and standard deviations (SD) or medians and interquartile ranges (IQR) as appropriate, and categorical variables using frequency and percentages. To assess inter-facility variability in screening uptake, timing, and methods, we compared proportions using χ² tests.

We used binary logistic regression to identify factors associated with being screened for GDM. We first conducted bivariable analyses to estimate crude odds ratios (COR) and then fitted a multivariable model to estimate adjusted odds ratios (AOR) with 95% confidence intervals (CI). Statistical significance was defined as p < 0.05. The Hosmer–Lemeshow goodness-of-fit test did not indicate evidence of poor fit (χ² = 7.73, df = 8, p = 0.461). Gravidity was not included in the multivariable model because of conceptual overlap and collinearity with parity.

### Ethical considerations

This study received ethical approval from the Institutional Review Board of the College of Health Sciences, Addis Ababa University (023/25/SPH) and Addis Ababa City Administration Health Bureau (RPO/992/25). A waiver of informed consent was granted as data were extracted from existing medical records, and all analyses used de-identified data. The names of included hospitals were also anonymized in the analysis to protect institutional confidentiality.

## Results

A total of 1,050 antenatal medical records (evenly sampled: 350 per facility) were abstracted. The mean (±SD) age of the women was 27.7 (± 4.9) years. Women were predominantly urban-residing (99.8%). About one-third of the women (29.4%) were pregnant for the first time (primigravida). Mean gestational age (GA) at first ANC contact was 19.5 (± 11.0) weeks, and the median (IQR) number of ANC visits was 6 (3,9). Table 1 shows baseline characteristics.

**Table 1:** Baseline socio-demographic and obstetric profile of antenatal care attendees at three public hospitals in Addis Ababa, Ethiopia, 2025 (N = 1,050).

Variable	Values*
Age, mean (±SD)	27.7 (± 4.9)
Number of pregnancies (gravidity), n (%)	
First pregnancy (primigravida)	309 (29.4)
Two or more pregnancies (multigravida)	741 (70.6)
Number of previous births (parity), n (%)	
No previous births (nulliparous)	376 (35.8)
One previous birth (primiparous)	360 (34.3)
Two or more previous births (multiparous)	314 (29.9)
Gestational age in weeks at first ANC visit, mean (±SD)	19.5 (± 11.0)
Number of ANC visits, n (%)	
≤3 visits	301 (28.7)
4-7 visits	331 (31.5)
≥8 visits	418 (39.8)

* Values are mean±SD, median (IQR) or n (%) as appropriate.

Abbreviations: SD = Standard deviations, IQR = inter-quartile range, n (%) = frequency (percentage), ANC = antenatal care.

### GDM screening uptake and guideline-adherence indicators

Of the 1050 antenatal records reviewed, 139 documented GDM screening, yielding an overall screening rate of 13.2%. Statistical comparison of screening rates across facilities demonstrated a significant difference (χ² = 30.27, p < 0.05) with uptake ranging from 5.1% to 18.0%.

Among screened women, the median gestational age at screening was 34 (30,37) weeks. Only 12/139 (8.6%) mothers were screened on time at 24–28 weeks with significant between-facility variation (χ² = 11.43, p < 0.05).

Regarding screening method used, the one-step 2-hr 75g OGTT was the predominant method used in 122 (87.8%) screened cases. Sixty-five (46.8%) screened mothers underwent more than one tests for screening. Guideline-concordant GDM screening (OGTT performed at 24–28 weeks’ gestation) was documented in 11/139 (7.9%) screened women.

Screening was almost exclusively risk-based (99.3%). Multiple risk factors that prompt GDM screening were recorded with the most frequent recorded reason for testing being polyhydramnios, 58 (41.7%), followed by advanced maternal age (≥35 years) in 34 (24.5%) and prior history of delivering macrosomic baby in 24 (17.3%) mothers. Additional documented indications for ordering testing were large for gestational age (LGA) in the index pregnancy (12.9%), history of GDM in previous pregnancy (9.4%), prior poor birth outcome (previous still birth, abortion, early neonatal death) (8.6%) and pre-pregnancy overweight/obesity (5.8%). Documentation of a universal-screening approach was present only in one (0.7%) case. Table 2 summarizes uptake and adherence to GDM screening.

**Table 2:** GDM screening uptake and guideline-adherence among women who underwent GDM screening at three public hospitals in Addis Ababa, Ethiopia, 2025 (N = 139).

Variable	Frequency (percentage)
Screened	139 (13.2)
Gestational age at GDM screening	
Before 24 weeks	15 (10.8)
24–28 weeks	12 (8.6)
29–32 weeks	36 (25.9)
>32 weeks	76 (54.7)
Screening methods used *	
OGTT	122 (87.8)
FBS	71 (51.1)
RBS or A1c	17 (12.3)
Guideline-concordant screening: OGTT at 24–28 weeks**	11 (7.9)

* more than one method was used for screening; ** This composite variable measures recommended timing and test modality, but does not fully capture guideline adherence as there was a limited universal offer of screening.

Abbreviations: n (%) = frequency (percentage), GDM = gestational diabetes mellitus, ANC = antenatal care, OGTT = oral glucose tolerance test, FBS = fasting blood sugar, RBS = random blood sugar, A1c = hemoglobin A1c.

### GDM yield and management

Among screened women, 57/139 (41.0%) were diagnosed with GDM. Screening prompted by polyhydramnios, prior GDM history, previous stillbirth history, and pregestational obesity/overweight was more frequently associated with GDM diagnosis compared with other triggers. GDM diagnosis was more frequent in those whose screening was triggered by polyhydramnios: 47.4% vs 37.8%, pregestational obesity/overweight: 10.5% vs 1.2%, prior GDM: 22.8% vs 0%, and previous still birth: 12.3% vs 0%. Of those diagnosed with GDM, half (49.1%) were initiated on pharmacotherapy and the remaining half were managed with lifestyle measures only.

### Factors associated with being screened for GDM

In multivariable logistic regression, the likelihood of GDM screening differed by facility, and several maternal profile (Table 3). Compared to facility A, women receiving ANC at facility B has markedly lower odds of being screened (AOR 0.23; 95% CI 0.12–0.42), whereas those at facility C had higher odds of screening (AOR 2.73; 95% CI 1.36–5.46).

**Table 3:** Factors associated with GDM screening among pregnant women attending three public hospitals in Addis Ababa, Ethiopia, 2025 (N = 1,050).

Explanatory variable	GDM screening		COR 95% CI	AOR 95% CI
	Not screened	Screened		
Facility, n(%)				
Facility A	292 (83.4)	58 (16.6)	1	1
Facility B	332 (94.9)	18 (5.1)	0.27 (0.16-0.47)	0.23 (0.12-0.42)***
Facility C	287 (82.0)	63 (18.0)	1.11 (0.75-1.64)	2.73 (1.36-5.46)**
Maternal age (years), mean ±SD	27.2 (±4.7)	30.9 (±5.3)	1.16 (1.12-1.20)	1.12 (1.07-1.16)***
Parity, n(%)				
Nulliparous	354 (94.1)	22 (5.9)	1	1
Primiparous	321 (89.2)	39 (10.8)	1.95 (1.14-3.37)	1.45 (0.82-2.56)
Multiparous	236 (75.2)	78 (24.8)	5.32 (3.22-8.77)	2.83 (1.63-4.92)***
Gestational age in weeks at first ANC visit, mean ±SD	20.5 (±10.8)	19.3 (±11.1)	1.01 (0.99-1.03)	0.95 (0.92-0.99)*
Number of ANC visits, median (IQR)	6 (3, 9)	5 (3, 8)	0.94 (0.88-1.99)	1.03 (0.93-1.14)

*p < 0.05; **p < 0.01; ***p < 0.001.

COR and AOR represent the odds of being screened for GDM.

Abbreviations: GDM = gestational diabetes mellitus, COR = crude odds ration, AOR = adjusted odds ration, ANC = antenatal care.

Screened women were, on average, older than unscreened women. Each additional year of maternal age was associated with a 12% increase in the odds of being screened (AOR 1.12 per additional year, 95% CI 1.07–1.16). Screening also showed a strong association with parity. Relative to nulliparous women, multiparous women had nearly three times higher adjusted odds of screening (AOR 2.83; 95% CI 1.63–4.92). In addition, earlier entry into ANC was independently associated with screening: each additional week delay in first ANC visit was associated with a 5% reduction in the odds of being screened (AOR 0.95, 95% CI 0.92–0.99).

More ANC visits showed a negative association with screening on crude comparisons but were not independently associated with screening in the adjusted model (AOR 1.03, 95% CI 0.93–1.14).

## Discussion

This study aimed to assess GDM screening uptake and adherence to guideline-recommended screening practice in three public hospitals in Addis Ababa, Ethiopia. This study adds to the limited implementation-focused evidence on GDM screening practice in Ethiopia and sub-Saharan Africa. The principal findings are that the overall uptake of GDM screening was very low, and the practice was late and risk-based varied substantially across included facilities.

The maternal profile of our study population is comparable to that of women receiving ANC in similar settings in the region. Our study population has a relatively young maternal age profile, high overall ANC contact but often late entry into care, and substantial proportions of multigravida and multiparous women, which was broadly consistent with reports from public hospitals in Addis Ababa [28] and similar urban centers across sub-Saharan Africa [20,21,29].

We found very low overall uptake of GDM screening (13.2%) with marked inter-facility variation (5.1–18%). Screening happened late with over half of the women screened after 32 weeks of gestation, and only fewer than one in ten tested on time. Besides, screening was almost entirely risk-based, triggered by overt clinical risk factors or complications. These findings are broadly consistent with reports from other sub-Saharan African settings that have documented low GDM screening coverage, late testing, and heavy reliance on risk-based approaches. Studies from Cameroon [20] and Tanzania [21] have reported screening rates below 30% and high proportions of undiagnosed GDM among unscreened women. Similar risk-factor–based screening practice has been described in a qualitative study from southern Ethiopia, where ANC providers reported that GDM screening was done based on risk factors rather than universally [24]. Our findings provide quantitative evidence that reinforces this picture. In contrast, many high-income countries reported universal or near-universal GDM screening coverage of above 90% [17–19].

The diagnostic yield among screened women was high (41%) in this study. The positivity rate is far higher than typical prevalence estimates from Ethiopia. A national systematic review and meta-analysis estimated a pooled GDM prevalence of approximately 12% [26], while facility-based studies using active research-based screening have reported higher estimates. For example, three studies in Southeast, South and central Ethiopia reported GDM estimates of 15.7% to 16.9% [28,30,31], while a more recent hospital-based cross-sectional study from Addis Ababa (including Gandhi Memorial Hospital) reported a GDM prevalence of 18.7% [32]. This reflects highly selective testing of women already perceived to be high risk or clinically complicated. This apparent discrepancy is also explained by differences in study design and denominators. Prior Ethiopian studies were prevalence surveys that offered screening around mid-gestation among randomly sampled pregnant women. We, however, enumerated all ANC clients in three hospitals and then examined GDM yield only among the small group who were actually tested in routine practice.

This study identified facility-level factors and maternal characteristics as factors independently associated with being screened for GDM. Where the mother received care and their obstetric profile (maternal age, parity, and timing of ANC initiation) predicted who is screened.

Facility type was an independent factor associated with being screened for GDM. While all three study sites operate within a similar urban health system, there was a marked inter-facility difference in their GDM screening practice. Although this study did not address why the screening practices varied by facility, the persistence of facility effect after adjusting for other measured variables suggests that facility level contexts shape screening uptake and timing. Importantly, implementation strategies to close this screening gap will require interventions tailored in emphasis and operationalization to the specific workflows of the facilities. Hence, future research should examine facility-level factors to inform context-sensitive implementation strategies.

Increasing maternal age was associated with an increased likelihood of screening. Multiparous women had also substantially higher odds of being screened than nulliparous women. This is consistent with risk-factor–based approaches to GDM screening and with qualitative evidence from Ethiopia [24] showing that providers tend to focus on women they perceive as at higher risk, particularly older and multiparous women.

Timing of ANC initiation was important factor. In our study, earlier entry into ANC was independently associated with higher odds of screening, whereas each additional week delay in first ANC visit reduced the likelihood of being screened. This finding aligns with the WHO’s recommendation and operational logic of GDM screening: ANC should start early to provide crucial early screenings [33], while those who enter care late may already be beyond that window or have limited remaining visits. In contrast, simply increasing the volume of care (the total number of ANC visits) was not independently associated with screening.

Several limitations of this study should be acknowledged. The analysis relies on retrospective documentation in clinical and laboratory records. Although triangulation with laboratory systems likely reduced misclassification of screening status, it is still possible that some screening tests could have been performed outside the hospital’s system, potentially underestimating screening rates. Selection bias is also possible because the study included only women accessing ANC in the participating hospitals. Because most women were never tested, and those who were tested were typically higher-risk and screened late, our data cannot be used to estimate the population prevalence of GDM in these hospitals or in Ethiopia in general. Lastly, because the facilities we selected are high-volume public referral settings, high patient load may have contributed to omission of recommended screening or incomplete documentation.

Despite these limitations, the results of this study contribute to the emerging body of implementation research on GDM screening in LMICs. Unlike most prior Ethiopian and regional studies that have assessed GDM prevalence and/or risk factors, we quantified GDM screening uptake, timing and methods of screening, and what triggers testing by triangulating EMR data with the laboratory information system. Hence, this study offers a picture of real-world screening practice in urban referral settings in Ethiopia.

Although this baseline audit was not designed to fully explain provider-level, facility-level and health-system-level determinants of GDM screening practice, the findings have direct implications for policymakers. The very low overall screening coverage indicates that endorsing the WHO GDM screening recommendations alone is insufficient to ensure implementation in routine ANC. On-time, universal GDM screening should therefore be enabled and monitored for implementation within existing ANC services. Besides, the near-exclusive reliance on risk-based testing demonstrates that current practice has not shifted from selective testing to universal offer to all pregnant women. Hence, policymakers should support a simplified screening package that reduces dependence on individual provider risk assessment. To this end, the findings from this study provide actionable baseline metrics for designing and evaluating implementation strategies to improve timely and guideline-concordant GDM screening in Ethiopia.

## Conclusion

In three high-volume public hospitals in Ethiopia, GDM screening uptake was found to be low, with late testing, and a selective rather than universal approach. This highlights an implementation gap more than a decade after national policy endorsement of systematic screening. Older maternal age, higher parity, earlier ANC initiation, and facility type were independent factors associated with being screened. These findings highlight a substantial implementation gap that supports the need for future research and operational strategies to translate universal GDM screening policy into routine ANC practice.

## Supporting information

S1 File De-identified minimal dataset underlying the study findings. (SAV)
