# Supplementary material for: Adherence to gestational diabetes screening guidelines: Retrospective audit in three public hospitals in Ethiopia
Source: PLoS One. 2026 Aug 25;21(8):e0357102. doi: 10.1371/journal.pone.0357102 (PMC13505934; doi:10.1371/journal.pone.0357102)
Supplement: Supplementary file 1 [file pone.0357102.s002.pdf]

Comments to Authors

Manuscript title: Adherence to gestational diabetes screening guidelines: retrospective audit in three public hospitals in Ethiopia.

- **Research Question:**
  - Does Adherence to gestational diabetes screening guidelines go with the primary outcome of the study (any GDM screening) mentioned in line 130 and results interpretations? Adherence to GDM screening guidelines was considered as secondary outcome?
- **Abstract**
  - a facility-based retrospective chart review vs A facility based cross-sectional study was conducted among pregnant women------ .Review is considered as data collection method but not a design
  - Indicate the measure of association used alongside logistic regression
  - The mean (±SD) maternal age of participants vs The mean (±SD) age of participants---
  - 24–28-week window vs 24–28-weeks window
  - In adjusted models, facility? Is it type facility? You better indicate the direction of the association.
  - What was the response variable? Was it being screened or Adherence to GDM screening guidelines? Results need to be interpreted based on the response variable.
  - In conclusion section, it is good to mention qualitatively factors associated with the response/outcome variable . ??????being screened or Adherence to GDM screening guidelines?
  - It is worthwhile to mention implementation strategies in accordance with the identified risks for not being screened
- **Introduction:**
  - Line 71-72: Early identification of GDM enables timely lifestyle and pharmacologic intervention, which has been shown to reduce adverse pregnancy outcomes -------check for grammar
  - What the prevalence of Adherence to gestational diabetes screening guidelines looks like in other LMICs particularly sub-Saharan Africa other than Ethiopia?
  - What are the factors associated with Adherence to GDM screening guidelines from previous studies? It is better to summarize in one paragraph.
  - Line 91-96: What is the actual gap? Is it lack of an implementation strategy to improve adherence to GDM screening guidelines? Paraphrase the gap in understandable way?
- **Methods**
  - Line 99: We conducted a retrospective cross-sectional study---- all cross-sectional studies are retrospective by nature so that the mention of “retrospective’ in design section is not supported.
  - Line 100-101: Why only public hospitals were considered?

- Line 107: The source population comprised pregnant women vs The source population comprised of pregnant women. The source and study population need to be explicitly described. Did you consider parity? When is GDM more common?
- Line 113: a single-population proportion formula with 95% confidence vs a single-population proportion formula with 95% confidence level?
- Line 113-114: Why highest GDM screening rate of 29.9% from a sub-Saharan African study (21) ? Why didn't you consider 50% when there was no previous study?
- Line 124: Data was collected from the EMRs vs Data were collected from the EMRs
- Where data quality issue was described in the methods section?
- Did you check model fitness and multicollinearity? If yes, describe appropriate methods used alongside their outputs in methods or result section.
- "The names of included hospitals 154 were also anonymized in the analysis to protect institutional confidentiality." How about in the study setting section?

- **Results:**

- What was the retrieval rate of the study?
- "Of the 1050 antenatal records reviewed, 139 documented GDM screening, yielding an overall screening rate of 13.2%." What proportion of the screening done was as per the GDM screening guideline?
- What was the data source for pre-pregnancy overweight/obesity (5.8%).???
- Line 214-215: there is a repetition?
- Line 200 & 215: Lack of consistency (Factors vs Determinants) ? For this study , the word "Factors" is more appropriate.
- Line 215-216: In cross tabulating the outcome variable (Table 3), the "Failure "category (Not screened) should appear first but(Being screened" category for the purpose of logistic regression interpretation. This could affect the computed crude odds ratios.
- Categories for variables maternal age , gestational age, and Number of ANC visits need to appear in the table in addition to mean and median values. Overall , the table lacks clarity.
- Number of astrix indicated in table 3 should be based on the strength of the association. But now they all are same (*) ??? Does it mean all the level of p-values is the same?

- **Discussion**

- The discussion section should start with the mention of the study objective and should follow the specific objectives of the study in turn.

- Line 221-222: This study provides the first multi-facility assessment of adherence to GDM screening guidelines in Ethiopia, and one of the very few such analyses from sub-Saharan Africa." However, from the result description, it seems that the study's main focus is "GDM screening uptake" than "Adherence to GDM screening guidelines"?? Any justification for that?
- Line 252, 255: This study identified facility-level factors and maternal characteristics as strong determinants GDM screening. Factors vs determinants??
- What could be the possible explanation for inter-facility variation in GDM screening yield being operating in a similar urban health system? Authors need to recommend further research otherwise?
- Line 241: "The diagnostic yield among screened women was high (41%) in this study." Does this have a direct relationship with the study objective?
- Line 252, 267: strong <u>determinants</u> of GDM screening? As commented elsewhere
- The policy implications of the findings need to be discussed. Overall, what do the findings of this study imply for policy makers and planners?

Conclusion:

- This section needs to be revisited in line with the specific objectives and key findings of the study
- In three high-volume public hospitals in Ethiopia, adherence to guideline-recommended GDM screening was found to be low. What level of adherence to guideline-recommended GDM screening was reported in this study?
- What is the possible recommendation for adherence to guideline-recommended GDM screening?

References:

- Ref.# 1, 23 and30 seem incomplete ,make them traceable using web citation(URL) and date accessed.
- Ref.#30: Organization WH should be written as " World Health Organization"
