# Supplementary material for: Adherence to gestational diabetes screening guidelines: Retrospective audit in three public hospitals in Ethiopia
Source: PLoS One. 2026 Aug 25;21(8):e0357102. doi: 10.1371/journal.pone.0357102 (PMC13505934; doi:10.1371/journal.pone.0357102)
Supplement: Supplementary file 2 [file pone.0357102.s003.docx]

Point-by-point response to editor and reviewers

Manuscript ID: PONE-D-26-12499

Title: Adherence to Gestational diabetes screening guidelines: retrospective audit in three public hospitals in Ethiopia.

Dear Editor,

We thank you and the reviewers for the detailed review of our manuscript and for the constructive feedback. In response, we have substantially revised the manuscript to improve conceptual consistency, methodological reporting, table clarity, and interpretation.

The main changes include: (1) clarifying that the primary outcome is any documented GDM screening, while guideline-adherence indicators are who is screened, when screened and with what screening tests – a composite indicator of guideline-concordant screening defined as OGTT performed at 24-28 weeks of gestation is added as the most plausible adherence indicator; (2) revising the design description (3) expanding the Methods to describe data sources, sample size, data quality procedures, testing methods, and model diagnostics; (4) revising the Results as per the feedback; (5) restructuring the Discussion to avoid overstatement, and add policy and system-level implications; and (6) revising tables, terminology consistency, and updating the references.

We have also prepared the required revised files: a manuscript with tracked changes, a clean revised manuscript, this point-by-point response, and a de-identified minimal dataset. Because line numbers change during formatting, the response below identifies the revised locations section in the clean manuscript version.

# Response to Academic Editor

|  | Editor comment | Response and revisions |
| --- | --- | --- |
| Comment E1: Clarification of Study Objective and Outcome | There is inconsistency throughout the manuscript regarding the main outcome: It is unclear whether the study focuses on: GDM screening uptake, or Adherence to GDM screening guidelines. Ensure consistency across all sections of the manuscript | We agree. We revised the title, abstract, introduction, methods, results, discussion, and conclusion to distinguish the primary outcome from secondary guideline adherence outcomes. The primary outcome is now consistently defined as any documented GDM screening. Guideline adherence is reported as a set of secondary implementation indicators: who is screened (screening uptake – ideally should have been all pregnant mothers universally), when screened (universal screening at 24–28 weeks of gestation is the ideal), and what screening test (OGTT is the preferred testing when feasible). We added a composite indicator of guideline-concordant screening defined as OGTT performed at 24-28 weeks of gestation. This resolves the inconsistency between “uptake” and “adherence.” |
| Comment E2: Methods Require Significant Improvement | Please revise the Methods section to improve clarity and transparency: Clearly describe the study design, Specify data sources (e.g., ANC registers, delivery records, EMRs), Provide a detailed explanation of: Sample size calculation, Sampling procedure and allocation across hospitals, Clarify eligibility criteria, particularly the inclusion of ANC attendees and potential bias, Describe the OGTT procedure and other diagnostic methods used, Provide details on: Statistical analysis and Model diagnostics (e.g., multicollinearity, model fit) | We substantially expanded the Methods. We described the study as a facility-based cross-sectional study; clearly explained EMR (has all antenatal, maternity and delivery records with same identifier - MRN) and laboratory information systems as data sources; clarified eligibility criteria; elaborated the sample-size calculation; described OGTT test procedure; and added data-quality sections, multicollinearity, and model-fit procedures. |
| Comment E3: results presentation | Report the record retrieval rate; Clarify the proportion of screening conducted according to guidelines; Justify denominators used in analysis; Improve tables (especially Table 3); Ensure clarity and consistency; Present categorical variables appropriately; Maintain consistent terminology throughout (e.g., “factors” vs “determinants”) | We addressed the comments to improve the results section. we clarified denominators for our analysis that screening uptake is calculated among all eligible records, while timing, method, documented indications, GDM diagnosis, and treatment are calculated among screened women because these variables are only applicable once screening occurs.  We added guideline-concordant screening (OGTT at 24–28 weeks) as an additional result. We revised tables 2 & 3 to improve clarity, and used the term “factors associated with screening” consistently. |
| Comment E4: strengthening the discussion | The discussion should be revised to: Avoid unsupported or overstated claims, Clearly reference and contextualize “recent studies”, Provide deeper interpretation of findings, particularly: Health system constraints, Provider-related factors, Facility-level variation. Expand on policy and practical implications | We updated the Discussion to begin with the study objective and qualified over-statements. We discussed the factors associated with screening as contextual association rather than causation. We also contextualized the ‘recent studies’ statement with clarity. Furthermore, we expanded policy/practice implications grounded in the study findings. |
| Comment E5: Bias and limitations | Please explicitly discuss: Potential selection bias (e.g., inclusion of ANC attendees only), Limitations of retrospective data and Impact of high patient volume in study facilities. | We expanded the Limitations section to explicitly discuss selection bias, limitations of retrospective documentation, and impact of high patient volume. |

# Response to Reviewer #1

|  | Reviewer comment | Response and revisions |
| --- | --- | --- |
| Comment R1.1: Research question | Does Adherence to gestational diabetes screening guidelines go with the primary outcome of the study (any GDM screening) mentioned in line 130 and results interpretations? Adherence to GDM screening guidelines was considered as secondary outcome? | We agree that this needs clarification. We now state that the primary outcome was any documented GDM screening (screening uptake). We now clarified guideline adherence as a set of indicators: who is screened (screening uptake – ideally should have been all pregnant mothers universally), when screened (ideally at 24–28 weeks of gestation for all pregnant mothers), and what screening test (OGTT as the preferred option when feasible). We also added a composite indicator of guideline-concordant screening defined as OGTT performed at 24-28 weeks of gestation as the most plausible composite secondary outcome. |
| Comment R1.2: abstract | “facility-based retrospective chart review” vs “facility-based cross-sectional study”; review is a data collection method, not a design. | We revised the study design wording accordingly (line 35). |
| Comment R1.3: Abstract | indicate the measure of association used alongside logistic regression. | Measure of association is added as ‘Logistic regression was used to estimate crude odds ratios (COR) and adjusted odds ratios (AOR) with 95% confidence intervals (CI) for factors associated with being screened’ (line 41-42) |
| Comment R1.4: Abstract | “The mean (±SD) maternal age of participants” vs “The mean (±SD) age of participants” And “24–28-week window” vs “24–28-weeks window” | Corrected both as recommended. |
| Comment R1.5: Abstract | in adjusted models, facility? Is it type facility? indicate direction of association. | Revised as ‘In adjusted analysis, facility site (hospital C), older maternal age, higher parity and earlier gestational age at first antenatal care were independently associated with higher odds of being screened.’ (line 52-54). |
| Comment R1.6: Abstract | What was the response variable? Was it being screened or Adherence to GDM screening guidelines? Results need to be interpreted based on the response variable | The response variable in regression was screened (yes/no). Adherence indicators are reported descriptively. This is clarified throughout the manuscript more clearly. |
| Comment R1.7: Conclusion | it is good to mention qualitatively factors associated with the response/outcome variable . ??????being screened or Adherence to GDM screening guidelines? It is worthwhile to mention implementation strategies in accordance with the identified risks for not being screened | Revised as recommended: ‘In three public hospitals in Addis Ababa, GDM screening uptake was low. The screening practice was also delayed, and risk-based. Being screened was associated with hospital site, older maternal age, higher parity, and earlier first antenatal care attendance. These findings suggest addressing patient-level factors and facility-tailored implementation strategies to achieve universal, timely GDM screening in routine antenatal care workflows’. |
| Comment R1.8: Introduction | grammar line 71–72. | Revised for grammar as “Early identification of GDM enables timely lifestyle and pharmacologic interventions that reduce adverse pregnancy outcomes...” (line 75-76) |
| Comment R1.9: Introduction | What the prevalence of Adherence to gestational diabetes screening guidelines looks like in other LMICs particularly sub-Saharan Africa other than Ethiopia? | We expanded the regional context paragraph summarizing evidence from sub-Saharan Africa (line 85-92). |
| Comment R1.10: Introduction | What are the factors associated with Adherence to GDM screening guidelines from previous studies? It is better to summarize in one paragraph | We added a paragraph summarizing SSA evidence on factors associated with GDM screening practice including late ANC booking, lack of standardized protocols, provider awareness, laboratory capacity, cost, supplies etc ( line 93-101). |
| Comment R1.11: Introduction | Line 91-96: What is the actual gap? Is it lack of an implementation strategy to improve adherence to GDM screening guidelines? Paraphrase the gap in understandable way? | Revised accordingly. The gap is now stated as the lack of routine-care implementation evidence (line 110) |
| Comment R1.12: Methods | Line 99: We conducted a retrospective cross-sectional study---- all cross-sectional studies are retrospective by nature so that the mention of “retrospective’ in design section is not supported | We revised to ‘cross sectional record review’(line 119). |
| Comment R1.13: Methods | Line 100-101: Why only public hospitals were considered? | Thank you for this important feedback. We agree that inclusion of private facilities would provide a more comprehensive picture of GDM screening practice. However, this study was the first phase of an implementation project designed to establish baseline metrics in selected high-volume public hospitals in Addis Ababa.  Public hospitals were selected because they are major platforms for ANC and referral maternity care and are the key public-sector settings where national ANC guidelines are expected to be implemented and scaled. We will try to incorporate this feedback in the next phases of our project as project feasibility and budget allows. |
| Comment R1.14: Methods | Line 107: The source population comprised pregnant women vs The source population comprised of pregnant women. The source and study population need to be explicitly described. Did you consider parity? When is GDM more common? | We corrected the grammar and clarified source vs study population (line 127-129). We did not consider parity as inclusion or exclusion criteria as universal screening principles recommend offering GDM testing for all pregnant women irrespective of parity or any other obstetric factors. |
| Comment R1.15: Methods | Line 113: a single-population proportion formula with 95% confidence vs a single-population proportion formula with 95% confidence level? | Corrected accordingly |
| Comment R1.16: Methods | Line 113-114: Why highest GDM screening rate of 29.9% from a sub-Saharan African study (21) ? Why didn’t you consider 50% when there was no previous study? | We used the highest available GDM screening coverage estimate from a relevant sub-Saharan African study (Tanzania) to ensure contextual relevance. Importantly, the final sample of 1,050 records exceeded the required sample size under both this prior estimate and a conservative 50% assumption. |
| Comment R1.17: Methods | Line 124: Data was collected from the EMRs vs Data were collected from the EMRs | grammar corrected in this section and subsequently throughout. |
| Comment R1.18: Methods | Where data quality issue was described in the methods section? | Thank you for this important comment. We agree that the original Methods section did not sufficiently describe the data quality procedures used. We have now added a dedicated subsection entitled “Data quality assurance” in the Methods section (line 161-170). |
| Comment R1.19: Methods | Did you check model fitness and multicollinearity? If yes, describe appropriate methods used alongside their outputs in methods or result section | thank you for this valuable comment. We assessed model fit for the logistic regression using the Hosmer–Lemeshow goodness-of-fit test. The test was not statistically significant (χ² = 7.73, df = 8, p = 0.461), indicating no evidence of poor model fit. We have added this information to the Methods sections of the revised manuscript. We also clarified that gravidity and parity were not entered together in the multivariable model because of conceptual overlap and potential collinearity (line 184-186). |
| Comment R1.20: Methods | “The names of included hospitals were also anonymized in the analysis to protect institutional confidentiality.” How about in the study setting section? | Thank you for this important observation. Because this is an implementation-focused study, we retained the names of the participating hospitals in the Study Setting section to provide relevant and rich context about the care settings in which this study was conducted. However, to reduce the risk of institutional reputational harm, or unintended ranking between hospitals, we report facility-specific results using masked labels (Hospital A, Hospital B, and Hospital C) and do not link these labels to hospital names in tables, figures, or narrative results.  In addition, the findings have already been shared with representatives from participating hospitals and national stakeholders during a dissemination workshop we conducted, and printed copies of the final publication will be shared with each participating center to support local care improvement. |
| Comment R1.21: Results | “Of the 1050 antenatal records reviewed, 139 documented GDM screening, yielding an overall  screening rate of 13.2%.” What proportion of the screening done was as per the GDM screening  guideline? | as we clarified in comment R1.1 above, we have updated primary and secondary outcomes more clearly. In the revised manuscript, we have stated that the primary outcome was GDM screening uptake, while adherence indicators as a composite of who is screened (universality), when screening occurs (timing), and what test is used (test modality).  We did not generate a single overall composite adherence score consisting of all adherence indicators because screening was overwhelmingly risk-based rather than universal. However, we added the most proximate composite marker (OGTT performed within 24-28 weeks) (line 213-214 and last row of table 2). Otherwise, we report each adherence component separately as shown in table 2. |
| Comment R1.22: Results | What was the retrieval rate of the study? | A total of 6,823 ANC visit records were comprehensively reviewed for eligibility across the three selected public hospitals during the study period (retrieval rate of 15.39%) |
| Comment R1.23: Results | “Of the 1050 antenatal records reviewed, 139 documented GDM screening, yielding an overall screening rate of 13.2%.” What proportion of the screening done was as per the GDM screening guideline? | We clarified the adherence indicators and added a composite marker of guideline-concordant screening: OGTT performed at 24-28 weeks of gestation as the most plausible marker of adherence, even though the screening was risk based (not universal) (see line 2 and table 2 last row.) |
| Comment R1.24: Results | What was the data source for pre-pregnancy overweight/obesity (5.8%).??? | All data we abstracted are from clinician-documented risk factors or indications for testing in the electronic record. The study team did not independently measure pre-pregnancy weight. |
| Comment R1.25: Results | repetition line 214–215. | Removed repetitive text. |
| Comment R1.26: Results | Line 200 & 215: Lack of consistency (Factors vs Determinants) ? For this study , the word “Factors”  is more appropriate | accepted and reworded to “factors associated with screening” throughout. |
| Comment R1.27: Results | Line 215-216: In cross tabulating the outcome variable (Table 3), the “Failure “category (Not screened) should appear first but(Being screened” category for the purpose of logistic regression interpretation. This could affect the computed crude odds ratios | We present the descriptive outcome columns in table 3 in the order Not screened followed by Screened. For the logistic regression analyses, all crude and adjusted odds ratios represent the odds of being screened for GDM. We clarified this in the table footnote. |
| Comment R1.28: Results | Number of astrix indicated in table 3 should be based on the strength of the association. But now they all are same (*) ??? Does it mean all the level of p-values is the same? | Revised accordingly. Asterisks are rewritten using the conventional notation: *p<0.05, **p<0.01, ***p<0.001. |
| Comment R1.29: Discussion | The discussion section should start with the mention of the study objective and should follow the  specific objectives of the study in turn | included this feedback and the discussion now begins by restating the study objective as in line 262-263. |
| Comment R1.30: Discussion | Line 221-222: This study provides the first multi-facility assessment of adherence to GDM screening guidelines in Ethiopia, and one of the very few such analyses from sub-Saharan Africa.”  However, from the result description, it seems that the study’s main focus is “GDM screening uptake”  than “Adherence to GDM screening guidelines”?? Any justification for that? | Revised. We clarify that the study assesses both uptake and guideline-adherence indicators to avoid implying that uptake alone equals adherence to guideline. |
| Comment R1.31: Discussion | What could be the possible explanation for inter-facility variation in GDM screening yield being operating in a similar urban health system? Authors need to recommend further research otherwise? | thank you for this comment as well. As we did not aim to address this question at this stage of our project, we stated the need for future research to examine facility-level factors for context-sensitive implementation strategies (line 304-307). |
| Comment R1.32: Discussion | Line 241: “The diagnostic yield among screened women was high (41%) in this study.” Does this have a direct relationship with the study objective? | it is true that this was not our primary objective. Diagnostic yield was a (secondary) descriptive finding among screened women. It was included because it informs interpretation of risk-based screening practice. |
| Comment R1.33: Discussion | The policy implications of the findings need to be discussed. Overall, what do the findings of this study imply for policy makers and planners? | Thank you for this important comment. We agree that the previous version did not sufficiently translate the findings into clear policy and planning implications. In the revised discussion, we have added a dedicated paragraph on policy and practice implications (line 336-346). |
| Comment R1.34: Conclusion | This section needs to be revisited in line with the specific objectives and key findings of the study. In three high-volume public hospitals in Ethiopia, adherence to guideline-recommended GDM screening was found to be low. What level of adherence to guideline-recommended GDM screening was reported in this study? What is the possible recommendation for adherence to guideline-recommended GDM screening? | conclusion section is revised and updated accordingly. |
| Comment R1.32: References | Ref.# 1, 23 and 30 seem incomplete , make them traceable using web citation(URL) and date  accessed. Ref.#30: Organization WH should be written as “ World Health Organization” | References are manually updated accordingly with updated reference lists including three new references added. |

# Response to Reviewer #2

|  | Reviewer comment | Response and revisions |
| --- | --- | --- |
| Comment R2.1: methods | The authors can describe the OGTT process for readers who may not be familiar with the procedure. Including the other testis available within the context. This is important because, the authors only make reference to the OGTT and yet in the data collection they include other tests as well. | We agree and added a description of the standard OGTT process (line 146-148). |
| Comment R2.2: limitation | I hope that the authors discuss selection of busy ANC as a limitation given that they are likely to omit certain critical procedures because of the high volume | Added to limitations. We now state that high patient volume in referral ANC clinics may contribute to missed procedures, or incomplete documentation (327-29). |
| Comment R2.3: methods | The authors have to demonstrate or describe to some detail how the sample size was derived and how this sample was distributed among the hospitals | We agree and now we expanded sample size section in methods. |
| Comment R2.4: methods | The study population and eligibility is a little bit confusing. If the authors used a retrospective data abstraction, they ought to describe clearly which register or medical record they used to obtained this information. Was it ANC, maternity etc records. The current description is confusing because it includes ANC, delivery etc. | Thank you for this important clarification. We have revised the Methods section to distinguish the source population, study population, eligibility criteria, and data sources (line 127-133). The primary data source was the medical record / EMR (which has all the antenatal care, maternity and delivery records with same EMR number) for women who attended ANC in the three study hospitals. Laboratory information systems were used to verify whether GDM-related tests had been performed to minimize misclassification of screening status. |
| Comment R2.5: methods | Using 'having attended ANC' as a selection criteria could be introducing potential biases into your study? "Non attendance of ANC" or "number of ANC attendance" could be an important factor in taking the test. No? Moreover, later in the discussions the authors discuss ANC attendance as an important factor. | Thank you for this important comment. We agree that restricting the study population to women who attended ANC in the participating hospitals introduces a potential selection bias, because women who did not attend ANC, attended ANC elsewhere, or had very limited contact with care may have different probabilities of receiving GDM screening. ANC attendance was included as an eligibility criterion because GDM screening is operationally delivered through ANC services. To assess whether screening was offered in routine care, women needed to have a documented ANC contact. We included this framing in the limitation section (line 324-325). |
| Comment R2.6: results | I am concerned about the denominator used to estimate Risk-based testing GDM. Why not use the total of 1050 as the denominator? | Documented indications for testing were recorded only when testing was ordered; therefore, we felt that the appropriate denominator for risk-based indications would be screened women. |
| Comment R2.7: results | (Line 176-184) It would appear like a sub group analysis. Was that the original idea/plan of the authors? | Thank you for this comment. This was a secondary descriptive analysis among women with documented GDM screening. Because universal screening was rarely practiced, reporting the risk-based indications for testing helped us explain what prompted screening in routine care. |
| Comment R2.8: discussion | Line 221 - 222 Is this not an overstatement? Does the literature search substantiate this assertion? | Thank you for this important comment. We agree that the original wording may have sounded overly definitive. To avoid overstatement, we now rewrote this first section of the discussion (line 262-266). At the time of our literature review, routine-care implementation of screening guidelines were not assessed in Ethiopia except one study (cited in the manuscript) which explored whether providers practice GDM screening or not in a qualitative study. We also identified only a small number of SSA studies that assessed screening uptake, timing, or test modality in routine settings, including the cited studies from Tanzania and Cameroon. |
| Comment R2.9: discussion | The authors make multiple references to 'recent studies' in lines 241-244 . The authors need to qualify these statements to guide the reader in what the message is | Thank you for this helpful comment. We agree that the phrase “recent studies” was too vague. In the revised Discussion, we have replaced this wording with a more specific synthesis of the cited literature (line 285-290). |
| Comment R2.10: discussion | In line 267 what is the evidence for the assertion that for every week delayed there is a less likely chance of taking GDM test? | This statement is based on our adjusted logistic regression finding that later gestational age at first ANC was associated with lower odds of screening (AOR 0.95, 95% CI 0.92–0.99) . We revised wording to avoid causal interpretation (line 313). |
| Comment R2.11: conclusion | This recommendation "improving universal and timely screening - to guide subsequent implementation research in Ethiopia and similar LMIC settings" (line 297) is what already exists within the policy documents for GDM screening, I presume? Are the authors not simply restating what already exist? | Revised the conclusion section (line 348-354). |
| Comment R2.12 | I think the findings highlight systemic issues related to the Human Resources, how much they know, what importance is attached to GDM and its severity, knowledge about the broader SDGs and major limitations for their achievements. These findings also raises the question of who is supervising the ANC clinics? The authors should discuss these findings from a systemic perspective. Otherwise, the associated factors per se provide very limited actionable insights | Thank you for this insightful comment. We agree that the associated factors identified in our quantitative analysis should not be interpreted narrowly as individual-level determinants alone. The screening practice likely reflect broader system-level issues. We have added a section in the discussion to highlight this as a cautionary note on interpretation. We also expanded the broader policy and planning implications (line 336-346).  We also would like to state that the current study was designed as a baseline quantitative audit and was not intended to fully identify the mechanisms underlying these system-level gaps. To address this, our project is conducting a complementary multilevel assessment using the Consolidated Framework for Implementation Research (CFIR) to explore provider-level, facility-level and health-system-level determinants of GDM screening practice. This will allow us to examine the very issues the reviewer raises. |

We thank the editor and reviewers again for their detailed and constructive feedback. We believe the revised manuscript has improved the conceptual clarity, statistical reporting, and policy relevance. We hope the revised version is now suitable for further consideration by PLOS ONE.

Sincerely,

Authors
