# Supplementary material for: Adherence to gestational diabetes screening guidelines: Retrospective audit in three public hospitals in Ethiopia
Source: PLoS One. 2026 Aug 25;21(8):e0357102. doi: 10.1371/journal.pone.0357102 (PMC13505934; doi:10.1371/journal.pone.0357102)
Supplement: Peer review history

# Peer review: Decision Letter 0

**DOI:** 10.1371/journal.pone.0357102.r001

**Authors:** Abera Lambebo Temamo

24 Apr 2026

PONE-D-26-12499Adherence to gestational diabetes screening guidelines: retrospective audit in three public hospitals in Ethiopia.PLOS One

Dear Dr. Amogne,

Thank you for submitting your manuscript to PLOS ONE. After careful consideration, we feel that it has merit but does not fully meet PLOS ONE’s publication criteria as it currently stands. Therefore, we invite you to submit a revised version of the manuscript that addresses the points raised during the review process.

Please submit your revised manuscript by Jun 08 2026 11:59PM. If you will need more time than this to complete your revisions, please reply to this message or contact the journal office at plosone@plos.org. When you're ready to submit your revision, log on to https://www.editorialmanager.com/pone/ and select the 'Submissions Needing Revision' folder to locate your manuscript file.

Please include the following items when submitting your revised manuscript:

- A letter that responds to each point raised by the academic editor and reviewer(s). You should upload this letter as a separate file labeled 'Response to Reviewers'.
- A marked-up copy of your manuscript that highlights changes made to the original version. You should upload this as a separate file labeled 'Revised Manuscript with Track Changes'.
- An unmarked version of your revised paper without tracked changes. You should upload this as a separate file labeled 'Manuscript'.

If you would like to make changes to your financial disclosure, please include your updated statement in your cover letter. Guidelines for resubmitting your figure files are available below the reviewer comments at the end of this letter.

If applicable, we recommend that you deposit your laboratory protocols in protocols.io to enhance the reproducibility of your results. Protocols.io assigns your protocol its own identifier (DOI) so that it can be cited independently in the future. For instructions see: https://journals.plos.org/plosone/s/submission-guidelines#loc-laboratory-protocols. Additionally, PLOS ONE offers an option for publishing peer-reviewed Lab Protocol articles, which describe protocols hosted on protocols.io. Read more information on sharing protocols at https://plos.org/protocols?utm_medium=editorial-email&utm_source=authorletters&utm_campaign=protocols.

As the corresponding author, your ORCID iD is verified in the submission system and will appear in the published article. PLOS supports the use of ORCID, and we encourage all coauthors to register for an ORCID iD and use it as well. Please encourage your coauthors to verify their ORCID iD within the submission system before final acceptance, as unverified ORCID iDs will not appear in the published article. *Only*  the individual author can complete the verification step; PLOS staff *cannot*  verify ORCID iDs on behalf of authors.

We look forward to receiving your revised manuscript.

Kind regards,

Abera Lambebo Temamo, Ph.D.

Academic Editor

PLOS One

Journal Requirements:

When submitting your revision, we need you to address these additional requirements.

1. Please ensure that your manuscript meets PLOS ONE's style requirements, including those for file naming. The PLOS ONE style templates can be found at

https://journals.plos.org/plosone/s/file?id=wjVg/PLOSOne_formatting_sample_main_body.pdf and

https://journals.plos.org/plosone/s/file?id=ba62/PLOSOne_formatting_sample_title_authors_affiliations.pdf

2. We note that your Data Availability Statement is currently as follows: “All relevant data are within the manuscript and its Supporting Information files”

Please confirm at this time whether or not your submission contains all raw data required to replicate the results of your study. Authors must share the “minimal data set” for their submission. PLOS defines the minimal data set to consist of the data required to replicate all study findings reported in the article, as well as related metadata and methods (https://journals.plos.org/plosone/s/data-availability#loc-minimal-data-set-definition).

For example, authors should submit the following data:

- The values behind the means, standard deviations and other measures reported;

- The values used to build graphs;

- The points extracted from images for analysis.

Authors do not need to submit their entire data set if only a portion of the data was used in the reported study.

If your submission does not contain these data, please either upload them as Supporting Information files or deposit them to a stable, public repository and provide us with the relevant URLs, DOIs, or accession numbers. For a list of recommended repositories, please see https://journals.plos.org/plosone/s/recommended-repositories.

If there are ethical or legal restrictions on sharing a de-identified data set, please explain them in detail (e.g., data contain potentially sensitive information, data are owned by a third-party organization, etc.) and who has imposed them (e.g., an ethics committee). Please also provide contact information for a data access committee, ethics committee, or other institutional body to which data requests may be sent. If data are owned by a third party, please indicate how others may request data access.

3. If the reviewer comments include a recommendation to cite specific previously published works, please review and evaluate these publications to determine whether they are relevant and should be cited. There is no requirement to cite these works unless the editor has indicated otherwise.

Additional Editor Comments:

Dear Authors,

Thank you for submitting your manuscript entitled “Adherence to Gestational Diabetes Screening Guidelines: Retrospective Audit in Three Public Hospitals in Ethiopia.” Your study addresses an important public health issue and contributes to the growing evidence on maternal health service delivery in low-resource settings.

After careful evaluation and consideration of the reviewers’ comments, we invite you to revise and resubmit your manuscript. While one reviewer suggested minor revisions, the other identified substantial methodological and interpretative concerns. Based on this, the overall decision is major revision.

Key Issues to Address

1. Clarification of Study Objective and Outcome

There is inconsistency throughout the manuscript regarding the main outcome:

It is unclear whether the study focuses on:

GDM screening uptake, or

Adherence to GDM screening guidelines

Ensure consistency across all sections of the manuscript.

2. Methods Require Significant Improvement

Please revise the Methods section to improve clarity and transparency:

Clearly describe the study design

Specify data sources (e.g., ANC registers, delivery records, EMRs)

Provide a detailed explanation of:

Sample size calculation

Sampling procedure and allocation across hospitals

Clarify eligibility criteria, particularly the inclusion of ANC attendees and potential bias

Describe the OGTT procedure and other diagnostic methods used

Provide details on:

Statistical analysis

Model diagnostics (e.g., multicollinearity, model fit)

3. Results Presentation

Report the record retrieval rate

Clarify the proportion of screening conducted according to guidelines

Justify denominators used in analysis

Improve tables (especially Table 3):

Ensure clarity and consistency

Present categorical variables appropriately

Maintain consistent terminology throughout (e.g., “factors” vs “determinants”)

4. Strengthening the Discussion

The discussion should be revised to:

Avoid unsupported or overstated claims

Clearly reference and contextualize “recent studies”

Provide deeper interpretation of findings, particularly:

Health system constraints

Provider-related factors

Facility-level variation

Expand on policy and practical implications

5. Bias and Limitations

Please explicitly discuss:

Potential selection bias (e.g., inclusion of ANC attendees only)

Limitations of retrospective data

Impact of high patient volume in study facilities

[Note: HTML markup is below. Please do not edit.]

Reviewers' comments:

Reviewer's Responses to Questions

**Comments to the Author**

1. Is the manuscript technically sound, and do the data support the conclusions?

The manuscript must describe a technically sound piece of scientific research with data that supports the conclusions. Experiments must have been conducted rigorously, with appropriate controls, replication, and sample sizes. The conclusions must be drawn appropriately based on the data presented.

Reviewer #1: Yes

Reviewer #2: Partly

**********

2. Has the statistical analysis been performed appropriately and rigorously?

Reviewer #1: No

Reviewer #2: No

**********

3. Have the authors made all data underlying the findings in their manuscript fully available?

The PLOS Data policy requires authors to make all data underlying the findings described in their manuscript fully available without restriction, with rare exception (please refer to the Data Availability Statement in the manuscript PDF file). The data should be provided as part of the manuscript or its supporting information, or deposited to a public repository. For example, in addition to summary statistics, the data points behind means, medians and variance measures should be available. If there are restrictions on publicly sharing data—e.g. participant privacy or use of data from a third party—those must be specified.

Reviewer #1: No

Reviewer #2: Yes

**********

4. Is the manuscript presented in an intelligible fashion and written in standard English?

PLOS ONE does not copyedit accepted manuscripts, so the language in submitted articles must be clear, correct, and unambiguous. Any typographical or grammatical errors should be corrected at revision, so please note any specific errors here.

Reviewer #1: Yes

Reviewer #2: No

**********

5. Review Comments to the Author

Please use the space provided to explain your answers to the questions above. You may also include additional comments for the author, including concerns about dual publication, research ethics, or publication ethics. (Please upload your review as an attachment if it exceeds 20,000 characters)

Reviewer #1: Comments to Authors

Manuscript title: Adherence to gestational diabetes screening guidelines: retrospective audit in three

public hospitals in Ethiopia.

• Research Question:

─ Does Adherence to gestational diabetes screening guidelines go with the primary outcome of the study (any GDM screening) mentioned in line 130 and results interpretations? Adherence to GDM screening guidelines was considered as secondary outcome?

• Abstract

─ a facility-based retrospective chart review vs A facility based cross-sectional study was conducted among pregnant women------ .Review is considered as data collection method but not a design

─ Indicate the measure of association used alongside logistic regression

─ The mean (±SD) maternal age of participants vs The mean (±SD) age of participants---

─ 24–28-week window vs 24–28-weeks window

─ In adjusted models, facility? Is it type facility? You better indicate the direction of the association.

─ What was the response variable? Was it being screened or Adherence to GDM screening guidelines? Results need to be interpreted based on the response variable.

─ In conclusion section, it is good to mention qualitatively factors associated with the response/outcome variable . ??????being screened or Adherence to GDM screening guidelines?

─ It is worthwhile to mention implementation strategies in accordance with the identified risks for not being screened

• Introduction:

─ Line 71-72: Early identification of GDM enables timely lifestyle and pharmacologic intervention, which has been shown to reduce adverse pregnancy outcomes -------check for grammar

─ What the prevalence of Adherence to gestational diabetes screening guidelines looks like in other LMICs particularly sub-Saharan Africa other than Ethiopia?

─ What are the factors associated with Adherence to GDM screening guidelines from previous studies? It is better to summarize in one paragraph.

─ Line 91-96: What is the actual gap? Is it lack of an implementation strategy to improve adherence to GDM screening guidelines? Paraphrase the gap in understandable way?

• Methods

─ Line 99: We conducted a retrospective cross-sectional study---- all cross-sectional studies are retrospective by nature so that the mention of “retrospective’ in design section is not supported.

─ Line 100-101: Why only public hospitals were considered?

─ Line 107: The source population comprised pregnant women vs The source population comprised of pregnant women. The source and study population need to be explicitly described. Did you consider parity? When is GDM more common?

─ Line 113: a single-population proportion formula with 95% confidence vs a single-population proportion formula with 95% confidence level?

─ Line 113-114: Why highest GDM screening rate of 29.9% from a sub-Saharan African study (21) ? Why didn’t you consider 50% when there was no previous study?

─ Line 124: Data was collected from the EMRs vs Data were collected from the EMRs

─ Where data quality issue was described in the methods section?

─ Did you check model fitness and multicollinearity? If yes, describe appropriate methods used alongside their outputs in methods or result section.

─ “The names of included hospitals 154 were also anonymized in the analysis to protect institutional confidentiality.” How about in the study setting section?

• Results:

─ What was the retrieval rate of the study?

─ “Of the 1050 antenatal records reviewed, 139 documented GDM screening, yielding an overall screening rate of 13.2%.” What proportion of the screening done was as per the GDM screening guideline?

─ What was the data source for pre-pregnancy overweight/obesity (5.8%).???

─ Line 214-215: there is a repetition?

─ Line 200 & 215: Lack of consistency (Factors vs Determinants) ? For this study , the word “Factors” is more appropriate.

─ Line 215-216: In cross tabulating the outcome variable (Table 3), the “Failure “category (Not screened) should appear first but(Being screened” category for the purpose of logistic regression interpretation. This could affect the computed crude odds ratios.

─ Categories for variables maternal age , gestational age, and Number of ANC visits need to appear in the table in addition to mean and median values. Overall , the table lacks clarity.

─ Number of astrix indicated in table 3 should be based on the strength of the association. But now they all are same (*) ??? Does it mean all the level of p-values is the same?

• Discussion

─ The discussion section should start with the mention of the study objective and should follow the specific objectives of the study in turn.

─ Line 221-222: This study provides the first multi-facility assessment of adherence to GDM screening guidelines in Ethiopia, and one of the very few such analyses from sub-Saharan Africa.” However, from the result description, it seems that the study’s main focus is “GDM screening uptake” than “Adherence to GDM screening guidelines”?? Any justification for that?

─ Line 252, 255: This study identified facility-level factors and maternal characteristics as strong determinants GDM screening. Factors vs determinants??

─ What could be the possible explanation for inter-facility variation in GDM screening yield being operating in a similar urban health system? Authors need to recommend further research otherwise?

─ Line 241: “The diagnostic yield among screened women was high (41%) in this study.” Does this have a direct relationship with the study objective?

─ Line 252, 267: strong determinants of GDM screening? As commented elsewhere

─ The policy implications of the findings need to be discussed. Overall, what do the findings of this study imply for policy makers and planners?

Conclusion:

─ This section needs to be revisited in line with the specific objectives and key findings of the study

─ In three high-volume public hospitals in Ethiopia, adherence to guideline-recommended GDM screening was found to be low. What level of adherence to guideline-recommended GDM screening was reported in this study?

─ What is the possible recommendation for adherence to guideline-recommended GDM screening?

References:

─ Ref.# 1, 23 and30 seem incomplete ,make them traceable using web citation(URL) and date accessed.

─ Ref.#30: Organization WH should be written as “ World Health Organization”

Reviewer #2: The authors can describe the OGTT process for readers who may not be familiar with the procedure. Including the other testis available within the context. This is important because, the authors only make reference to the OGTT and yet in the data collection they include other tests as well.

I hope that the authors discuss selection of busy ANC as a limitation given that they are likely to omit certain critical procedures because of the high volume.

The authors have to demonstrate or describe to some detail how the sample size was derived and how this sample was distributed among the hospitals. The study population and eligibility is a little bit confusing. If the authors used a retrospective data abstraction, they ought to describe clearly which register or medical record they used to obtained this information. Was it ANC, maternity etc records. The current description is confusing because it includes ANC, delivery etc.

Using 'having attended ANC' as a selection criteria could be introducing potential biases into your study? "Non attendance of ANC" or "number of ANC attendance" could be an important factor in taking the test. No? Moreover, later in the discussions the authors discuss ANC attendance as an important factor.

I am concerned about the denominator used to estimate Risk-based testing GDM. Why not use the total of 1050 as the denominator?

(Line 176-184) It would appear like a sub group analysis. Was that the original idea/plan of the authors?

In table 3, was it your original idea to make comparisons between the three hospitals?

Line 221 - 222 Is this not an overstatement? Does the literature search substantiate this assertion?

The authors make multiple references to 'recent studies' in lines 241-244 . The authors need to qualify these statements to guide the reader in what the message is.

In line 267 what is the evidence for the assertion that for every week delayed there is a less likely chance of taking GDM test? This recommendation "improving universal and timely screening - to guide subsequent implementation research in Ethiopia and similar LMIC settings" (line 297) is what already exists within the policy documents for GDM screening, I presume? Are the authors not simply restating what already exist?

I think the findings highlight systemic issues related to the Human Resources, how much they know, what importance is attached to GDM and its severity, knowledge about the broader SDGs and major limitations for their achievements. These findings also raises the question of who is supervising the ANC clinics? The authors should discuss these findings from a systemic perspective. Otherwise, the associated factors per se provide very limited actionable insights.

**********

6. PLOS authors have the option to publish the peer review history of their article (what does this mean?). If published, this will include your full peer review and any attached files.

If you choose “no”, your identity will remain anonymous but your review may still be made public.

**Do you want your identity to be public for this peer review?**  For information about this choice, including consent withdrawal, please see our Privacy Policy.

Reviewer #1: **Yes:**  Gudina Egata(PhD)

Reviewer #2: **Yes:**  Richard Mangwi Ayiasi

**********

[NOTE: If reviewer comments were submitted as an attachment file, they will be attached to this email and accessible via the submission site. Please log into your account, locate the manuscript record, and check for the action link "View Attachments". If this link does not appear, there are no attachment files.]

To ensure your figures meet our technical requirements, please review our figure guidelines: https://journals.plos.org/plosone/s/figures

You may also use PLOS’s free figure tool, NAAS, to help you prepare publication quality figures: https://journals.plos.org/plosone/s/figures#loc-tools-for-figure-preparation.

NAAS will assess whether your figures meet our technical requirements by comparing each figure against our figure specifications.

Attachment Submitted filename: Comments to Authors.pdf

# Author comment: Author response to Decision Letter 1

**DOI:** 10.1371/journal.pone.0357102.r002

8 Jun 2026

Point-by-point response to editor and reviewers

Manuscript ID: PONE-D-26-12499

Title: Adherence to Gestational diabetes screening guidelines: retrospective audit in three public hospitals in Ethiopia.

Dear Editor,

We thank you and the reviewers for the detailed review of our manuscript and for the constructive feedback. In response, we have substantially revised the manuscript to improve conceptual consistency, methodological reporting, table clarity, and interpretation.

The main changes include: (1) clarifying that the primary outcome is any documented GDM screening, while guideline-adherence indicators are who is screened, when screened and with what screening tests – a composite indicator of guideline-concordant screening defined as OGTT performed at 24-28 weeks of gestation is added as the most plausible adherence indicator; (2) revising the design description (3) expanding the Methods to describe data sources, sample size, data quality procedures, testing methods, and model diagnostics; (4) revising the Results as per the feedback; (5) restructuring the Discussion to avoid overstatement, and add policy and system-level implications; and (6) revising tables, terminology consistency, and updating the references.

We have also prepared the required revised files: a manuscript with tracked changes, a clean revised manuscript, this point-by-point response, and a de-identified minimal dataset. Because line numbers change during formatting, the response below identifies the revised locations section in the clean manuscript version.

Response to Academic Editor

Editor comment Response and revisions

Comment E1: Clarification of Study Objective and Outcome There is inconsistency throughout the manuscript regarding the main outcome: It is unclear whether the study focuses on: GDM screening uptake, or Adherence to GDM screening guidelines. Ensure consistency across all sections of the manuscript We agree. We revised the title, abstract, introduction, methods, results, discussion, and conclusion to distinguish the primary outcome from secondary guideline adherence outcomes. The primary outcome is now consistently defined as any documented GDM screening. Guideline adherence is reported as a set of secondary implementation indicators: who is screened (screening uptake – ideally should have been all pregnant mothers universally), when screened (universal screening at 24–28 weeks of gestation is the ideal), and what screening test (OGTT is the preferred testing when feasible). We added a composite indicator of guideline-concordant screening defined as OGTT performed at 24-28 weeks of gestation. This resolves the inconsistency between “uptake” and “adherence.”

Comment E2: Methods Require Significant Improvement Please revise the Methods section to improve clarity and transparency: Clearly describe the study design, Specify data sources (e.g., ANC registers, delivery records, EMRs), Provide a detailed explanation of: Sample size calculation, Sampling procedure and allocation across hospitals, Clarify eligibility criteria, particularly the inclusion of ANC attendees and potential bias, Describe the OGTT procedure and other diagnostic methods used, Provide details on: Statistical analysis and Model diagnostics (e.g., multicollinearity, model fit) We substantially expanded the Methods. We described the study as a facility-based cross-sectional study; clearly explained EMR (has all antenatal, maternity and delivery records with same identifier - MRN) and laboratory information systems as data sources; clarified eligibility criteria; elaborated the sample-size calculation; described OGTT test procedure; and added data-quality sections, multicollinearity, and model-fit procedures.

Comment E3: results presentation Report the record retrieval rate; Clarify the proportion of screening conducted according to guidelines; Justify denominators used in analysis; Improve tables (especially Table 3); Ensure clarity and consistency; Present categorical variables appropriately; Maintain consistent terminology throughout (e.g., “factors” vs “determinants”) We addressed the comments to improve the results section. we clarified denominators for our analysis that screening uptake is calculated among all eligible records, while timing, method, documented indications, GDM diagnosis, and treatment are calculated among screened women because these variables are only applicable once screening occurs.

We added guideline-concordant screening (OGTT at 24–28 weeks) as an additional result. We revised tables 2 & 3 to improve clarity, and used the term “factors associated with screening” consistently.

Comment E4: strengthening the discussion The discussion should be revised to: Avoid unsupported or overstated claims, Clearly reference and contextualize “recent studies”, Provide deeper interpretation of findings, particularly: Health system constraints, Provider-related factors, Facility-level variation. Expand on policy and practical implications We updated the Discussion to begin with the study objective and qualified over-statements. We discussed the factors associated with screening as contextual association rather than causation. We also contextualized the ‘recent studies’ statement with clarity. Furthermore, we expanded policy/practice implications grounded in the study findings.

Comment E5: Bias and limitations Please explicitly discuss: Potential selection bias (e.g., inclusion of ANC attendees only), Limitations of retrospective data and Impact of high patient volume in study facilities. We expanded the Limitations section to explicitly discuss selection bias, limitations of retrospective documentation, and impact of high patient volume.

Response to Reviewer #1

Reviewer comment Response and revisions

Comment R1.1: Research question Does Adherence to gestational diabetes screening guidelines go with the primary outcome of the study (any GDM screening) mentioned in line 130 and results interpretations? Adherence to GDM screening guidelines was considered as secondary outcome? We agree that this needs clarification. We now state that the primary outcome was any documented GDM screening (screening uptake). We now clarified guideline adherence as a set of indicators: who is screened (screening uptake – ideally should have been all pregnant mothers universally), when screened (ideally at 24–28 weeks of gestation for all pregnant mothers), and what screening test (OGTT as the preferred option when feasible). We also added a composite indicator of guideline-concordant screening defined as OGTT performed at 24-28 weeks of gestation as the most plausible composite secondary outcome.

Comment R1.2: abstract “facility-based retrospective chart review” vs “facility-based cross-sectional study”; review is a data collection method, not a design. We revised the study design wording accordingly (line 35).

Comment R1.3: Abstract indicate the measure of association used alongside logistic regression. Measure of association is added as ‘Logistic regression was used to estimate crude odds ratios (COR) and adjusted odds ratios (AOR) with 95% confidence intervals (CI) for factors associated with being screened’ (line 41-42)

Comment R1.4: Abstract “The mean (±SD) maternal age of participants” vs “The mean (±SD) age of participants” And “24–28-week window” vs “24–28-weeks window” Corrected both as recommended.

Comment R1.5: Abstract in adjusted models, facility? Is it type facility? indicate direction of association. Revised as ‘In adjusted analysis, facility site (hospital C), older maternal age, higher parity and earlier gestational age at first antenatal care were independently associated with higher odds of being screened.’ (line 52-54).

Comment R1.6: Abstract What was the response variable? Was it being screened or Adherence to GDM screening guidelines? Results need to be interpreted based on the response variable The response variable in regression was screened (yes/no). Adherence indicators are reported descriptively. This is clarified throughout the manuscript more clearly.

Comment R1.7: Conclusion it is good to mention qualitatively factors associated with the response/outcome variable . ??????being screened or Adherence to GDM screening guidelines? It is worthwhile to mention implementation strategies in accordance with the identified risks for not being screened Revised as recommended: ‘In three public hospitals in Addis Ababa, GDM screening uptake was low. The screening practice was also delayed, and risk-based. Being screened was associated with hospital site, older maternal age, higher parity, and earlier first antenatal care attendance. These findings suggest addressing patient-level factors and facility-tailored implementation strategies to achieve universal, timely GDM screening in routine antenatal care workflows’.

Comment R1.8: Introduction grammar line 71–72. Revised for grammar as “Early identification of GDM enables timely lifestyle and pharmacologic interventions that reduce adverse pregnancy outcomes...” (line 75-76)

Comment R1.9: Introduction What the prevalence of Adherence to gestational diabetes screening guidelines looks like in other LMICs particularly sub-Saharan Africa other than Ethiopia? We expanded the regional context paragraph summarizing evidence from sub-Saharan Africa (line 85-92).

Comment R1.10: Introduction What are the factors associated with Adherence to GDM screening guidelines from previous studies? It is better to summarize in one paragraph We added a paragraph summarizing SSA evidence on factors associated with GDM screening practice including late ANC booking, lack of standardized protocols, provider awareness, laboratory capacity, cost, supplies etc ( line 93-101).

Comment R1.11: Introduction Line 91-96: What is the actual gap? Is it lack of an implementation strategy to improve adherence to GDM screening guidelines? Paraphrase the gap in understandable way? Revised accordingly. The gap is now stated as the lack of routine-care implementation evidence (line 110)

Comment R1.12: Methods Line 99: We conducted a retrospective cross-sectional study---- all cross-sectional studies are retrospective by nature so that the mention of “retrospective’ in design section is not supported We revised to ‘cross sectional record review’(line 119).

Comment R1.13: Methods Line 100-101: Why only public hospitals were considered? Thank you for this important feedback. We agree that inclusion of private facilities would provide a more comprehensive picture of GDM screening practice. However, this study was the first phase of an implementation project designed to establish baseline metrics in selected high-volume public hospitals in Addis Ababa.

Public hospitals were selected because they are major platforms for ANC and referral maternity care and are the key public-sector settings where national ANC guidelines are expected to be implemented and scaled. We will try to incorporate this feedback in the next phases of our project as project feasibility and budget allows.

Comment R1.14: Methods Line 107: The source population comprised pregnant women vs The source population comprised of pregnant women. The source and study population need to be explicitly described. Did you consider parity? When is GDM more common? We corrected the grammar and clarified source vs study population (line 127-129). We did not consider parity as inclusion or exclusion criteria as universal screening principles recommend offering GDM testing for all pregnant women irrespective of parity or any other obstetric factors.

Comment R1.15: Methods Line 113: a single-population proportion formula with 95% confidence vs a single-population proportion formula with 95% confidence level? Corrected accordingly

Comment R1.16: Methods Line 113-114: Why highest GDM screening rate of 29.9% from a sub-Saharan African study (21) ? Why didn’t you consider 50% when there was no previous study? We used the highest available GDM screening coverage estimate from a relevant sub-Saharan African study (Tanzania) to ensure contextual relevance. Importantly, the final sample of 1,050 records exceeded the required sample size under both this prior estimate and a conservative 50% assumption.

Comment R1.17: Methods Line 124: Data was collected from the EMRs vs Data were collected from the EMRs grammar corrected in this section and subsequently throughout.

Comment R1.18: Methods Where data quality issue was described in the methods section? Thank you for this important comment. We agree that the original Methods section did not sufficiently describe the data quality procedures used. We have now added a dedicated subsection entitled “Data quality assurance” in the Methods section (line 161-170).

Comment R1.19: Methods Did you check model fitness and multicollinearity? If yes, describe appropriate methods used alongside their outputs in methods or result section thank you for this valuable comment. We assessed model fit for the logistic regression using the Hosmer–Lemeshow goodness-of-fit test. The test was not statistically significant (χ² = 7.73, df = 8, p = 0.461), indicating no evidence of poor model fit. We have added this information to the Methods sections of the revised manuscript. We also clarified that gravidity and parity were not entered together in the multivariable model because of conceptual overlap and potential collinearity (line 184-186).

Comment R1.20: Methods “The names of included hospitals were also anonymized in the analysis to protect institutional confidentiality.” How about in the study setting section? Thank you for this important observation. Because this is an implementation-focused study, we retained the names of the participating hospitals in the Study Setting section to provide relevant and rich context about the care settings in which this study was conducted. However, to reduce the risk of institutional reputational harm, or unintended ranking between hospitals, we report facility-specific results using masked labels (Hospital A, Hospital B, and Hospital C) and do not link these labels to hospital names in tables, figures, or narrative results.

In addition, the findings have already been shared with representatives from participating hospitals and national stakeholders during a dissemination workshop we conducted, and printed copies of the final publication will be shared with each participating center to support local care improvement.

Comment R1.21: Results “Of the 1050 antenatal records reviewed, 139 documented GDM screening, yielding an overall

screening rate of 13.2%.” What proportion of the screening done was as per the GDM screening

guideline? as we clarified in comment R1.1 above, we have updated primary and secondary outcomes more clearly. In the revised manuscript, we have stated that the primary outcome was GDM screening uptake, while adherence indicators as a composite of who is screened (universality), when screening occurs (timing), and what test is used (test modality).

We did not generate a single overall composite adherence score consisting of all adherence indicators because screening was overwhelmingly risk-based rather than universal. However, we added the most proximate composite marker (OGTT performed within 24-28 weeks) (line 213-214 and last row of table 2). Otherwise, we report each adherence component separately as shown in table 2.

Comment R1.22: Results What was the retrieval rate of the study? A total of 6,823 ANC visit records were comprehensively reviewed for eligibility across the three selected public hospitals during the study period (retrieval rate of 15.39%)

Comment R1.23: Results “Of the 1050 antenatal records reviewed, 139 documented GDM screening, yielding an overall screening rate of 13.2%.” What proportion of the screening done was as per the GDM screening guideline? We clarified the adherence indicators and added a composite marker of guideline-concordant screening: OGTT performed at 24-28 weeks of gestation as the most plausible marker of adherence, even though the screening was risk based (not universal) (see line 2 and table 2 last row.)

Comment R1.24: Results What was the data source for pre-pregnancy overweight/obesity (5.8%).??? All data we abstracted are from clinician-documented risk factors or indications for testing in t

Attachment Submitted filename: Response to Reviewers.docx

# Peer review: Decision Letter 1

**DOI:** 10.1371/journal.pone.0357102.r003

**Authors:** Zhaoxia Liang

13 Aug 2026

Adherence to gestational diabetes screening guidelines: retrospective audit in three public hospitals in Ethiopia.

PONE-D-26-12499R1

Dear Dr. Amogne,

We’re pleased to inform you that your manuscript has been judged scientifically suitable for publication and will be formally accepted for publication once it meets all outstanding technical requirements.

Within one week, you’ll receive an e-mail detailing the required amendments. When these have been addressed, you’ll receive a formal acceptance letter and your manuscript will be scheduled for publication.

An invoice will be generated when your article is formally accepted. Please note, if your institution has a publishing partnership with PLOS and your article meets the relevant criteria, all or part of your publication costs will be covered. Please make sure your user information is up-to-date by logging into Editorial Manager at Editorial Manager® and clicking the ‘Update My Information' link at the top of the page. For questions related to billing, please contact billing support.

If your institution or institutions have a press office, please notify them about your upcoming paper to help maximize its impact. If they’ll be preparing press materials, please inform our press team as soon as possible -- no later than 48 hours after receiving the formal acceptance. Your manuscript will remain under strict press embargo until 2 pm Eastern Time on the date of publication. For more information, please contact onepress@plos.org.

Kind regards,

Zhaoxia Liang

Academic Editor

PLOS One

Additional Editor Comments (optional):

Dear Authors,

I have reviewed your revised manuscript together with the comments from the two reviewers. Both reviewers have carefully assessed your revisions and recommend acceptance.

You have adequately addressed all the concerns raised during the initial review. This retrospective audit provides valuable insights regarding adherence to gestational diabetes screening guidelines in public hospitals in Ethiopia.

I am pleased to accept your manuscript for publication in PLOS ONE.

Congratulations.

Reviewers' comments:

Reviewer's Responses to Questions

**Comments to the Author**

1. If the authors have adequately addressed your comments raised in a previous round of review and you feel that this manuscript is now acceptable for publication, you may indicate that here to bypass the “Comments to the Author” section, enter your conflict of interest statement in the “Confidential to Editor” section, and submit your "Accept" recommendation.

Reviewer #1: All comments have been addressed

Reviewer #2: All comments have been addressed

**********

2. Is the manuscript technically sound, and do the data support the conclusions?

The manuscript must describe a technically sound piece of scientific research with data that supports the conclusions. Experiments must have been conducted rigorously, with appropriate controls, replication, and sample sizes. The conclusions must be drawn appropriately based on the data presented.

Reviewer #1: Yes

Reviewer #2: Yes

**********

3. Has the statistical analysis been performed appropriately and rigorously?

Reviewer #1: Yes

Reviewer #2: I Don't Know

**********

4. Have the authors made all data underlying the findings in their manuscript fully available?

The PLOS Data policy requires authors to make all data underlying the findings described in their manuscript fully available without restriction, with rare exception (please refer to the Data Availability Statement in the manuscript PDF file). The data should be provided as part of the manuscript or its supporting information, or deposited to a public repository. For example, in addition to summary statistics, the data points behind means, medians and variance measures should be available. If there are restrictions on publicly sharing data—e.g. participant privacy or use of data from a third party—those must be specified.

Reviewer #1: Yes

Reviewer #2: Yes

**********

5. Is the manuscript presented in an intelligible fashion and written in standard English?

PLOS ONE does not copyedit accepted manuscripts, so the language in submitted articles must be clear, correct, and unambiguous. Any typographical or grammatical errors should be corrected at revision, so please note any specific errors here.

Reviewer #1: Yes

Reviewer #2: Yes

**********

6. Review Comments to the Author

Please use the space provided to explain your answers to the questions above. You may also include additional comments for the author, including concerns about dual publication, research ethics, or publication ethics. (Please upload your review as an attachment if it exceeds 20,000 characters)

Reviewer #1: Dear authors , than for entertaining all question to the desired level. Your critical analysis of comments has brought the manuscript to the present level.

Reviewer #2: Authors have addressed all comments. The manuscript reads well and the flow is coherent. The methodology was revised to give clarity. The discussions and conclusions are aligned to the findings. Please check the reference list. Some of them require formatting e.g. reference from WHO

**********

7. PLOS authors have the option to publish the peer review history of their article (what does this mean?). If published, this will include your full peer review and any attached files.

If you choose “no”, your identity will remain anonymous but your review may still be made public.

**Do you want your identity to be public for this peer review?**  For information about this choice, including consent withdrawal, please see our Privacy Policy.

Reviewer #1: **Yes:**  Gudina Egata

Reviewer #2: **Yes:**  Richard Mangwi Ayiasi

**********

# Editor report: Acceptance letter

**DOI:** 10.1371/journal.pone.0357102.r004

**Authors:** Zhaoxia Liang

PONE-D-26-12499R1

PLOS One

Dear Dr. Amogne,

I'm pleased to inform you that your manuscript has been deemed suitable for publication in PLOS One. Congratulations! Your manuscript is now being handed over to our production team.

At this stage, our production department will prepare your paper for publication. This includes ensuring the following:

* All references, tables, and figures are properly cited

* All relevant supporting information is included in the manuscript submission,

* There are no issues that prevent the paper from being properly typeset

You will receive further instructions from the production team, including instructions on how to review your proof when it is ready. Please keep in mind that we are working through a large volume of accepted articles, so please give us a few days to review your paper and let you know the next and final steps.

Lastly, if your institution or institutions have a press office, please let them know about your upcoming paper now to help maximize its impact. If they'll be preparing press materials, please inform our press team within the next 48 hours. Your manuscript will remain under strict press embargo until 2 pm Eastern Time on the date of publication. For more information, please contact onepress@plos.org.

You will receive an invoice from PLOS for your publication fee after your manuscript has reached the completed accept phase. If you receive an email requesting payment before acceptance or for any other service, this may be a phishing scheme. Learn how to identify phishing emails and protect your accounts at https://explore.plos.org/phishing.

If we can help with anything else, please email us at customercare@plos.org.

Thank you for submitting your work to PLOS One and supporting open access.

Kind regards,

PLOS ONE Editorial Office Staff

on behalf of

Dr. Zhaoxia Liang

Academic Editor

PLOS One
